# Using human umbilical cord mesenchymal stem cells combined with allogenic platelet-rich fibrin membrane for the treatment of dual limb ischemia in an elderly patient: A case report

**DOI:** 10.1097/MD.0000000000025068

**Published:** 2021-03-12

**Authors:** Wendong Ju, Simao Fu, Jun Huang, Bishuang Li, Li Wang, Minmin Zheng, Haojie Song, Quanyong Li, Qiaoyun Zhou, Meixing He

**Affiliations:** a Department of Hematology Rheumatology; b Department of Pediatrics; c Department of Electrocardiology; d Department of Stomatology, Boai Hospital of Zhongshan, Zhongshan City, China.

**Keywords:** atherosclerosis, lower limb ischemia, mesenchymal stem cells, platelet-rich fibrin

## Abstract

**Rationale:**

To describe the clinical effects of human umbilical cord mesenchymal stem cells (UCMSCs) combined with allogenic platelet-rich fibrin (PRF) for the treatment of lower limb ischemia in an elderly patient.

**Patient concerns:**

The patient was a 93-year-old Chinese woman with bilateral foot gangrene and ulcers lasting for 6 months. She had a prior history of Behcet's disease.

**Diagnoses:**

The admitting diagnosis for this episode was atherosclerosis bilateral limb ischemia.

**Interventions:**

First, treatment consisting of immunosuppressants, anticoagulation, antiplatelets, and anti-microbials were instituted. A UCMSC suspension was administered intravenously and injected into the lower limbs twice. An allogenic PRF membrane was externally applied 15 times over the lower limbs.

**Outcomes:**

The patient's pain improved and the 6 ulcers healed.

**Lessons:**

The combination of UCMSCs with a PRF membrane for the treatment of lower limb ischemia in an elderly patient is effective and safe. More and larger trials are needed before incorporating this therapy into mainstream treatment.

## 1 Introduction

Conventional treatment to manage limb ischemia has not been the most satisfactory.^[1]^ For patients that are frail with significant comorbidities, the risks associated with surgery may be too high;^[2]^ the patient may end up with ulcers or amputated limbs and eventually death. Hence, an alternative treatment may be needed to preserve limb function. In this case report, we aim to describe the use of human umbilical cord mesenchymal stem cells (UCMSCs) combined with allogenic platelet-rich fibrin (PRF) for the treatment of lower limb ischemia in an elderly patient.

## 2 Description of case

**Figure 1:**
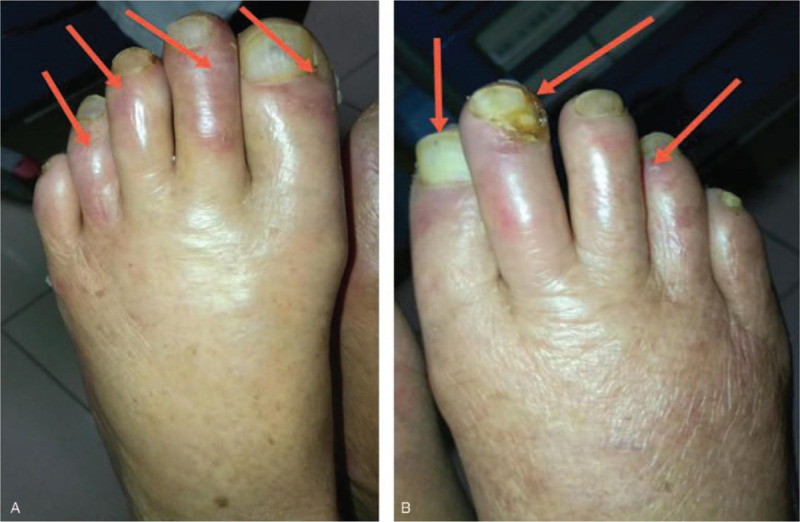
Foot ulcer before treatment. (A). Left foot of patient (preadmission). Arrows indicate the toes with ulcers. (B). Right foot of patient (preadmission). Arrows indicate the toes with ulcers.

A 93-year-old Chinese woman was referred to our hospital when she presented with bilateral foot gangrene and ulcers lasting for 6 months. She had a medical history of Behcet's disease, pulmonary infarction, myocardial infarction, and Parkinson's disease. Routine regional treatment and anticoagulant therapy showed no efficacy. On physical examination after admission on April 2019, the patient's pulse was irregular, both limbs were swollen, and the temperature on both limbs was low, with 7 toes showing signs of gangrene and 6 of the toes having ulcers with pus-like exudate. The sizes of the ulcers were uneven; the largest was 3.2 × 2.5 cm with an erythematous surrounding (Fig. 1a and b) and a weak pulse was felt on both dorsalis pedis. The laboratory test results were 94 g/L for hemoglobin, and 1.00 μg/ml for the D-dimer. The electrocardiogram revealed atrial fibrillation. Bilateral limb arterial Doppler ultrasound revealed a plaque in the left limb with a size of 5.7 × 1.5 mm and another in the right limb with a size of 7.0 × 3.0 mm. Stenosis was observed in more than 70% of the right limb. The admitting diagnosis for this episode was atherosclerosis bilateral limb ischemia.

**Figure 2:**
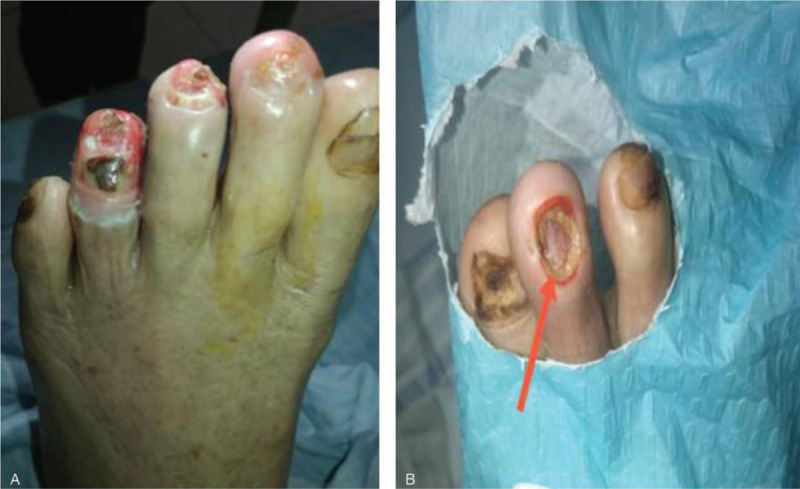
Wound debridement with demolin powder. (A). Left foot of patient after wound debridement. (B). Close-up of left foot of patient after wound debridement. The arrows indicate the bone of the patient.

**Figure 3:**
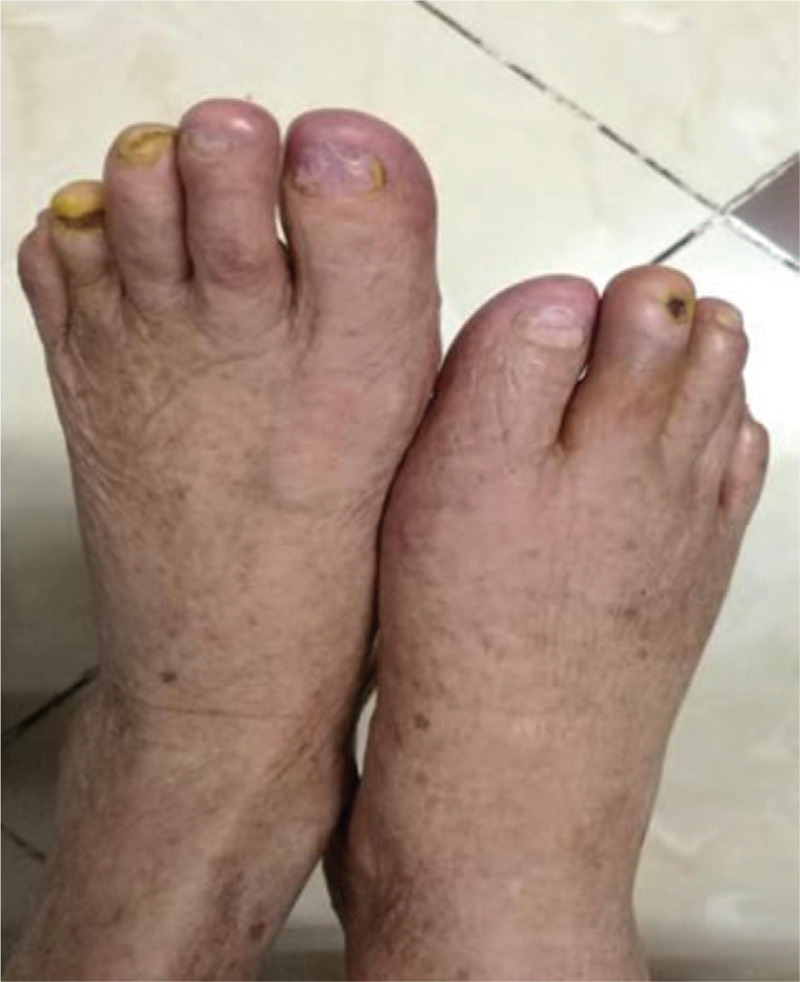
Fully recovered feet of patient.

**Figure 4:**
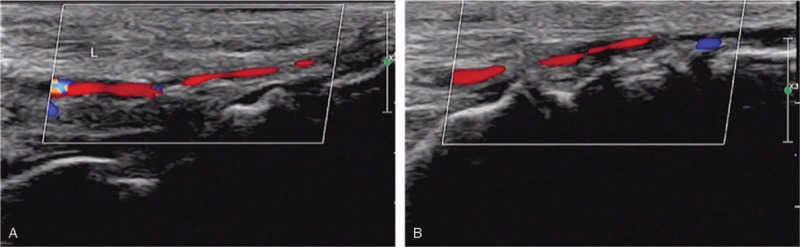
Color Doppler ultrasound images of dorsalis pedis artery. (A). Color Doppler flow image of left dorsalis pedis artery. (B). Color Doppler flow image of right dorsalis pedis artery.

The patient was given antimicrobials, immunosuppressants (methotrexate), anticoagulants (low molecular weight heparin), and antiplatelets (Clopidogrel). After topical anesthesia, a scalpel was used to remove necrotic tissue and tissue removal was stopped when healthy tissue was seen (i.e., with mild bleeding). Normal saline was used to irrigate the wound afterward. The wound was dried (Fig. 2a and b), treated with Demolin Powder, and dressed with silver ion alginate dressing. The leg was then wrapped in sterile dressing. After approval from the hospital ethical committee and informed consent from the patient, we started the patient on USCMCs with PRF therapy. We injected the USCMCs (manufactured by Guangzhou Celera Stem Cell Company, 100 ml with 5 × 10^7^ cells, 95% survival) into the vein and directly into the wound simultaneously. For the intravenous route, we slowly injected the stem cell suspension (3 × 10^7^ cells) into the vein. For the direct inoculation route, after 30 minutes of topical anesthesia with compound lidocaine ointment in the operating theatre, we disinfected the wound and directly injected the stem cell suspension (2 × 10^7^ cells) intramuscularly along the affected limb's artery and its branches using a 1 ml syringe. Each injection volume was 0.5 ml, with a distance of 1.0 to 3.0 cm between each injection site, focusing on the area surrounding the wound. The allogenic PRF (PRF-A) was donated by the patients’ relatives (healthy with no infections). From the relatives, 36 to 48 ml of venous blood was drawn, separated into dry tubes (6 ml each), and centrifuged at 1500 revolutions per minute for 14 minutes. After removing the supernatant, the middle segment rich in platelets and leukocyte fibrin glue (about 0.8 × 1.5–3 cm) was extruded in a sterile pot and a PRF film was prepared for later use. After a second surgical debridement procedure, the PRF film was applied over the various ulcers. A dry dressing was used to remove excess water and a silver ion alginate dressing was applied with sterile bandages. After routinely changing the dressing, the PRF film on the wound surface was checked. UCMSCs were injected into the vein every 2 weeks. After the 8th application of the PRF film, the bone-exposed regions were completely covered with granulation tissue. After the 15th application of the PRF film, the wounds were completely healed (Fig. 3). There were no significant adverse events except for the aggravation of the swelling in the bilateral lower limbs after the first injection of UCMSCs. After the first UCMSC injection, the patient felt much better, with improved alertness and appetite as well as the resolution of atrial fibrillation on the electrocardiogram. Arterial Doppler ultrasound revealed improvement of the stenosis. There was no significant improvement in plaque formation and stenosis of the lower extremity arteries. However, the diastolic blood flow velocity of the bilateral dorsalis pedis arteries increased (Fig. 4), and the right ankle brachial index improved from 0.82 to 0.9. Hand tremors and myotonia in Parkinson's disease were significantly reduced (Unified Parkinson's Disease Rating Scale (UPDRS) II+III partial scores decreased from 74 to 48). After 1.5 years of follow-up, the patient reported no recurrence of bilateral limb pain and ulcers.

## 3 Discussion

This is the first case report describing the use of both UCMSCs and PRF in lower limb ischemia management, as either modality was used separately in most studies to date. Intravenous injection and local application of UCMSCs were also key to ensuring good blood circulation as well as the combination of UCMSCs and PRF.

Unlike bone marrow stem cells, UCMSCs have a painless collection process and fast self-renewal properties.^[3]^ UCMSCs can automatically home in on and live in an ischemic environment. They release a variety of vascular growth factors through autocrine and paracrine effects to promote angiogenesis after ischemia and promote the recovery of blood flow in patients with ischemic diseases.^[4]^ According to a review by Ding et al, no cases of stem cell transplantation-related toxicity have been reported. A trial conducted by Suzdaltseva et al revealed that locally delivered allogenic UCMSCs can improve wound healing in chronic non-healing wounds, which further supports our case report.^[5]^

PRF is an improved formulation of platelet-rich plasma that is easier to use and does not require extrinsic anticoagulation or extra activation factors like thrombin.^[6]^ PRF is a promising therapeutic for wound healing because it is angiogenic and can control the immune system, recruit circulating stem cells, and ensure undisturbed wound healing.^[7]^ Trials conducted by Sharma et al using PRF and collagen dressing as a palatal bandage for wound healing revealed that PRF and collagen dressing significantly improved wound healing and reduced the patient's pain and discomfort.^[1]^ This provides additional evidence for our study.

In conclusion, we successfully demonstrated the use of UCMSCs and PRF for the treatment of lower limb ischemia in an elderly patient. UCMSCs and PRF-A may have a synergistic role in the treatment. In this case, after treatment with UCMSCs, the symptoms of Parkinson's disease improved, and the UPDRS score decreased significantly. It may be related to the differentiation of HuMSCs into astrocytes number of nerve cells and transforming into dopaminergic neurons in the brain. Sun et al^[8]^ reported that after carotid artery UCMSC transplantation, the UPDRS score and Webster score improved significantly, but the ankylosis state did not. UPDRS score decreased significantly.^[8]^ Although promising, there is a need for larger trials to confirm the efficacy and safety of this experimental therapy.

## Author contributions

**Data curation:** Li Wang.

**Formal analysis:** Li Wang.

**Investigation:** Jun Huang.

**Methodology:** Bishuang Li, Minmin Zheng, Haojie Song, Quanyong Li.

**Project administration:** Simao Fu.

**Resources:** Meixing He.

**Supervision:** Simao Fu.

**Validation:** Quanyong Li, Qiaoyun Zhou.

**Writing – original draft:** Wendong Ju.

**Writing – review & editing:** Wendong Ju, Jun Huang.
